# First report of natural infection of *Anopheles gambiae s.s.* and* Anopheles coluzzii* by *Wolbachia* and Microsporidia in Benin: a cross-sectional study

**DOI:** 10.1186/s12936-024-04906-1

**Published:** 2024-03-11

**Authors:** Minassou Juvénal Ahouandjinou, Arthur Sovi, Aboubakar Sidick, Wilfried Sewadé, Come Zinsou Koukpo, Saïd Chitou, Linda Towakinou, Bruno Adjottin, Steve Hougbe, Filémon Tokponnon, Germain Gil Padonou, Martin Akogbéto, Louisa A. Messenger, Razaki A. Ossè

**Affiliations:** 1 Centre de Recherche Entomologique de Cotonou, Cotonou, Benin; 2 Faculté d’Agronomie, Université de Parakou, Parakou, Benin; 3 Disease Control Department, Faculty of Infectious and Tropical Diseases, London School of Hygiene and Tropical Medicine, London, UK; 4 Biology Department, VERG Laboratories, Hacettepe University, Beytepe-Ankara, Turkey; 5 Ecole Polytechnique d’Abomey-Calavi, Université d’Abomey-Calavi, Abomey-Calavi, Benin; 6 Faculté des Sciences et Techniques, Université d’Abomey-Calavi, Abomey-Calavi, Benin; 7 Department of Environmental and Occupational Health, School of Public Health, University of Nevada, Las Vegas, NV 89154 USA; 8 Parasitology and Vector Biology Laboratory (UNLV PARAVEC Lab), School of Public Health, University of Nevada, Las Vegas, NV USA; 9 Ecole de Gestion et d’Exploitation des Systèmes d’Elevage, Université Nationale d’Agriculture, Kétou, Benin

**Keywords:** *Wolbachia*, *Microsporidia*, *Anopheles coluzzii*, *Anopheles gambiae*

## Abstract

**Background:**

Recently, bacterial endosymbiont, including *Wolbachia* and Microsporidia were found to limit the infection of *Anopheles* mosquitoes with *Plasmodium falciparum*. This study aimed to investigate the natural presence of key transmission-blocking endosymbionts in *Anopheles gambiae* and *Anopheles coluzzii* in Southern Benin*.*

**Methods:**

The present study was conducted in seven communes (Cotonou, Porto-Novo, Aguégués, Ifangni, Pobè Athiémé, and Grand-Popo) of Southern Benin. *Anopheles* were collected using indoor/outdoor Human Landing Catches (HLCs) and Pyrethrum Spray Catches (PSCs). Following morphological identification, PCR was used to identify *An. gambiae *sensu lato (*s.l.)* to species level and to screen for the presence of both *Wolbachia* and Microsporidia*. Plasmodium falciparum* sporozoite infection was also assessed using ELISA*.*

**Results:**

Overall, species composition in *An. gambiae s.l.* was 53.7% *An. coluzzii*, while the remainder was *An. gambiae *sensu stricto (*s.s.*)*.* Combined data of the two sampling techniques revealed a mean infection prevalence with *Wolbachia* of 5.1% (95% CI 0.90–18.6) and 1.3% (95% CI 0.07–7.8) in *An. gambiae s.s.* and *An. coluzzii*, respectively. The mean infection prevalence with Microsporidia was 41.0% (95% CI 25.9–57.8) for *An. gambiae s.s*. and 57.0% (95% CI 45.4–67.9) for *An. coluzzii. Wolbachia* was only observed in Ifangni, Pobè, and Cotonou, while Microsporidia was detected in all study communes. Aggregated data for HLCs and PSCs showed a sporozoite rate (SR) of 0.80% (95% CI 0.09–2.87) and 0.69% (95% CI 0.09–2.87) for *An. gambiae* and *An. coluzzii*, respectively, with a mean of 0.74% (95% CI 0.20–1.90). Of the four individual mosquitoes which harboured *P. falciparum*, none were also infected with *Wolbachia* and one contained Microsporidia.

**Conclusions:**

The present study is the first report of natural infections of field-collected *An. gambiae s.l.* populations from Benin with *Wolbachia* and Microsporidia. Sustained efforts should be made to widen the spectrum of bacteria identified in mosquitoes, with the potential to develop endosymbiont-based control tools; such interventions could be the game-changer in the control of malaria and arboviral disease transmission.

**Supplementary Information:**

The online version contains supplementary material available at 10.1186/s12936-024-04906-1.

## Background

Malaria is an infectious disease caused by a parasite of the *Plasmodium* genus with half of the global population at risk of this disease. In 2020, there were globally 247 million cases, and 619,000 deaths due to malaria [1]. Sub-Saharan Africa, where *Plasmodium falciparum* remains the most prevalent malaria parasite, bears the greatest global burden of disease [2]. The cornerstones of malaria vector control have been long-lasting insecticidal nets (LLINs) and indoor residual spraying (IRS) and have averted 1.5 billion malaria cases and 7.6 million malaria deaths, with these interventions accounting for 68% and 10% of these achievements, respectively [3]. With the scale-up of these interventions, the disease burden in Africa is expected to be significantly reduced by 2030. However, widespread insecticide resistance [4] and changes in vector behavior [5] may sabotage elimination in the upcoming decades. For that, biological control tools such as exploitation of *Wolbachia*, *Spiroplasma*, and Microsporidia endosymbionts that can be used alone or in combination with insecticide based-tools, have been developed to improve the control of vector-borne diseases, including malaria [6–9]. These bacteria have a large array of interactions including mutualism, commensalism, and parasitism within their hosts [10]. *Wolbachia* can colonize certain mosquito populations, and impact pathogen development, thereby reducing their infection and transmission potential [7, 8, 11]. Laboratory experiments have shown an absence of dengue virus infection in populations of *Aedes aegypti* artificially infected with *Wolbachia* [7, 12]. In addition, other laboratory trials showed that some *Wolbachia* strains impede infection of *Anopheles* vectors with *Plasmodium* species [13–16], making it an alternative option for malaria control. However, evidence of an impact of *Wolbachia* infection on malaria transmission at the community level is still scarce [13, 14]. It has long been assumed that *Wolbachia* is absent from natural populations of *Anopheles* [17]. It is only recently that studies have reported that *Anopheles gambiae *sensu stricto (*s.s*.), *Anopheles coluzzii* and *Anopheles arabiensis* can be found naturally infected by *Wolbachia* in Burkina Faso and Mali [18–20] and *Anopheles moucheti* and *Anopheles demeilloni* have been reported infected by *Wolbachia* in Cameroon, Kenya and the Democratic Republic of the Congo, with evidence of the capacity to induce cytoplasmic incompatibility [15]. Negative correlations between the presence of *Wolbachia* and development of *Plasmodium* has been demonstrated in *An. gambiae* in Mali and *An. coluzzii* in Burkina Faso [20, 21]. This supports the need for developing new vector control tools based on *Wolbachia*-*Anopheles* interactions.

The first report of Microsporidia in *An. arabiensis* was in Kenya, where Microsporidia infected mosquitoes were unable to be infected with *P. falciparum* [22]. The presence of this endosymbiont in wild vector populations, warrants screening for it in other endemic regions in Africa.

To progress the development of endosymbiont-based malaria control tools, it is important to continue identifying, and characterizing the native range of endosymbiont-infected *Anopheles* vector populations. The present study conducted in Southern Benin aims to identify the natural presence of *Wolbachia* and Microsporidia in *Anopheles gambiae s.l.,* the main malaria vector in this region.

## Methods

### Study area

**Fig. 1:**
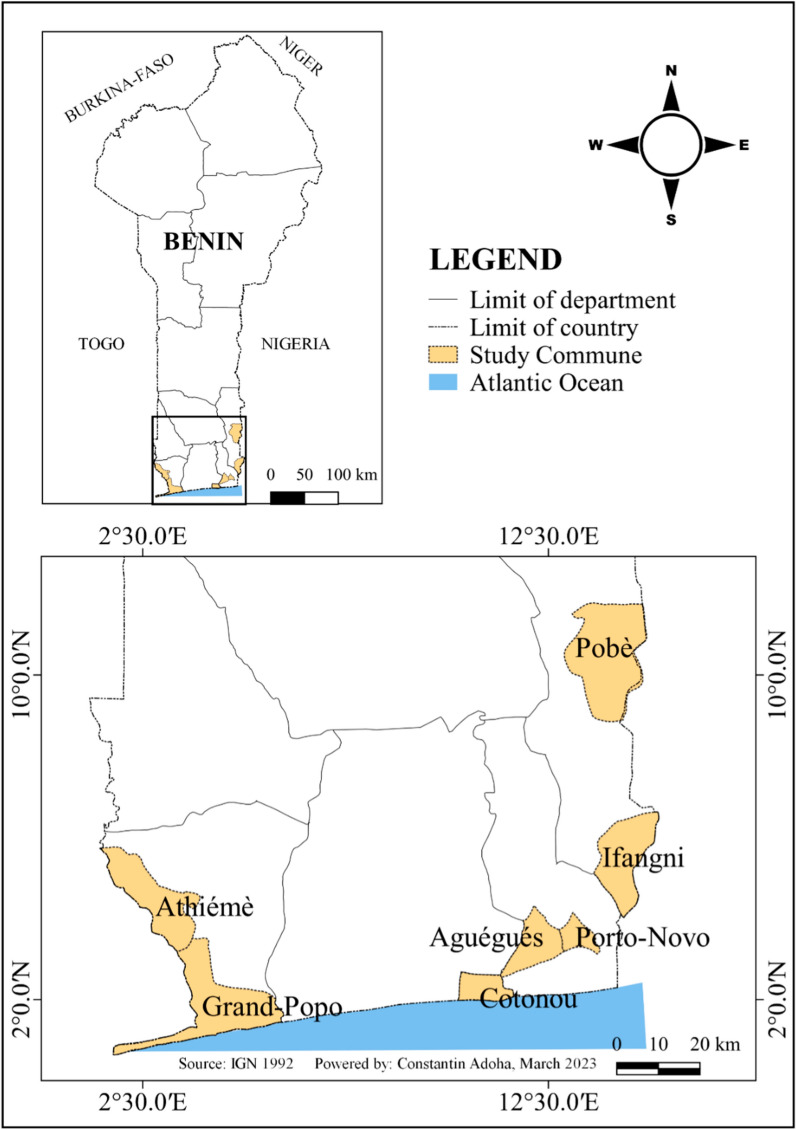
Map of the study area

The present study was conducted in September–October 2022 in seven communes **(**Cotonou, Porto-Novo, Aguégués, Ifangni, Pobè, Athiémé, and Grand-Popo) of Southern Benin (Fig. 1), characterized by a subequatorial climate with two wet (April to July, and September to October), and two dry (November to March, and July to August) seasons. The highest temperatures in the area were between 28 °C and 32 °C, and the lowest between 23 °C and 26 °C. The annual rainfall in the area was approximately 1245 mm, and the main malaria vector species were *An. coluzzii* and *An. gambiae s.s.* [23].

### Mosquito collections

The present study occurred in September–October 2022. In each study commune, adult mosquitoes were collected using human landing catches (HLCs). In each of two randomly selected houses, two (one indoors and one outdoors) trained collectors were positioned between 08:00 p.m. and 01:00 a.m. and replaced by two others between 01:00 a.m. and 06:00 a.m. Using mouth aspirators and flashlights, they collected all mosquitoes that attempted to bite their lower limbs.

In addition, collection of mosquitoes was also performed using pyrethrum spray catches (PSCs), performed early in the morning in 10 houses selected at random in each surveyed commune. This collection technique consisted of laying white sheets on the floor, closing all openings in the rooms, and spraying aerosol insecticides indoors. After 10–15 min, all indoor resting mosquitoes that fell on the sheets, after insecticidal exposure, were collected using forceps and petri-dishes.

Adult mosquitoes collected through the two sampling techniques were morphologically identified using a binocular loupe, according to the taxonomic keys of Gillies and Coetzee [24], and individually stored on silicagel at − 20 °C for further molecular analyses.

### Molecular analyses

#### Detection of *P. falciparum* sporozoite infection and molecular species identification

All specimens of *An. gambiae *sensu lato (*s.l.*) collected with HLCs and PSCs were analysed using ELISA-CSP to identify *P. falciparum* sporozoite infection [25]. Molecular species identification was performed in all caught specimens of *An. gambiae s.l.* using the protocol of Santolamazza et al*.* [26].

### Identification of the presence of endosymbiont

Overall, a total of 118 pools, each containing 5 specimens of either *An. gambiae* or *An. coluzzii* were formed. The genomic DNA of these pools was extracted using DNeasy Blood and Tissue kits (Qiagen, France), following the manufacturer instructions:

- Microsporidia
  The following primers: MB18SF (CGCCGGCCGTGAAAAATTTA) and MB18SR (CCTTGGACGTGGGGAGCTATC) were used to detect Microsporidia in *An. gambiae s.l.* [22]*.* Each PCR reaction consisted of a final volume of 12.5 µl with 120 ng/µl of DNA, 1 X Hot Start Taq (Thermo Scientific), and 0.3 µM of each primer. The conditions used were an initial denaturation at 95 °C for 5 min, 35 denaturation cycles at 95 °C for 1 min, hybridization at 62 °C for 90 s and an extension at 72 °C for a further 60 s. The final elongation was carried out at 72 °C for 5 min.
- Molecular detection of *Wolbachia*
  120 ng/µl of DNA was used to amplify a region of the 16S rDNA of *Wolbachia* using a nested PCR approach, which is specific for natural *Wolbachia* Anga infections in *An. gambiae s.l.* [21]. The primer pairs specific to *Wolbachia* Anga were Forward: 5ʹ-CATACCTATTCGAAGGGATAG-3ʹ; and Reverse: 5ʹ-AGCTTCGAGTGAAACCAATTC-3ʹ [27], which were used for the first reaction. The conditions for this amplification were: 5 min at 95 °C, followed by 45 cycles of 45 s at 95 °C, 45 s at 60 °C, 1 min at 72 °C, and 5 min at 72 °C. This was followed by a second amplification step using 0.3 µM of each primer—Forward: 5′-GAAGGGATAGGGTCGGTCG-3′ and Reverse: 5′-CAATTCCCATGCGTGGACG-3′ in a final reaction volume of 15.5 µl composed of 120 ng/µl of DNA and 1 × Hot Start Taq buffer (Thermo Scientific), using the following conditions: 15 min at 95 °C, followed by 35 cycles of 15 s at 95 °C, 25 s at 66 °C, 1 min at 72 °C, and 5 min at 72 °C [20]. Amplified fragments of 412 bp corresponding to *Wolbachia* Anga were confirmed by electrophoresis on 2% agarose gels.

## Results

### Mosquito species composition

**Fig. 2:**
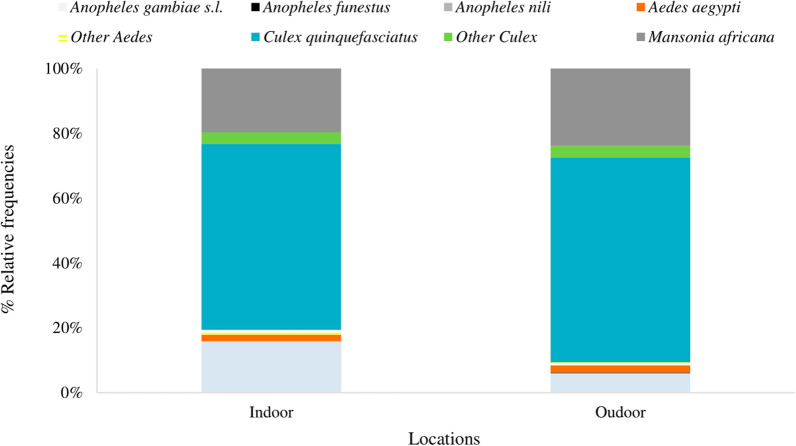
Mosquito species composition in the study area (HLC data)

Overall, a total of 6225 mosquitoes were collected using HLCs, with a higher ratio (77.8%, n = 4841) outdoors. Indoors, the most frequent mosquito species were *Culex quinquefasciatus* (57.4%), followed by *Mansonia africana* (19.7%), and *An. gambiae s.l.* (15.8%). The same trend was observed outdoors. Other mosquito species such as *Anopheles funestus*, *Ae. aegypti*, and other *Culex* spp. were also collected but at lower frequencies (< 4%) (Fig. 2).

The same trend was observed with PSCs that collected 305 mosquitoes, with *Cx. quinquefasciatus* being the most frequent mosquito species (64.6%), followed by *Mansonia africana* (14.8%), and *An. gambiae s.l.* (9.2%) (Additional file 1: Table S1).

Of 538 specimens of *An. gambiae* *s.l.* collected through the two sampling techniques and molecularly speciated, 53.7% (n = 289) were *An. coluzzii*, while the rest was *An. gambiae s.s.*

**Fig. 3:**
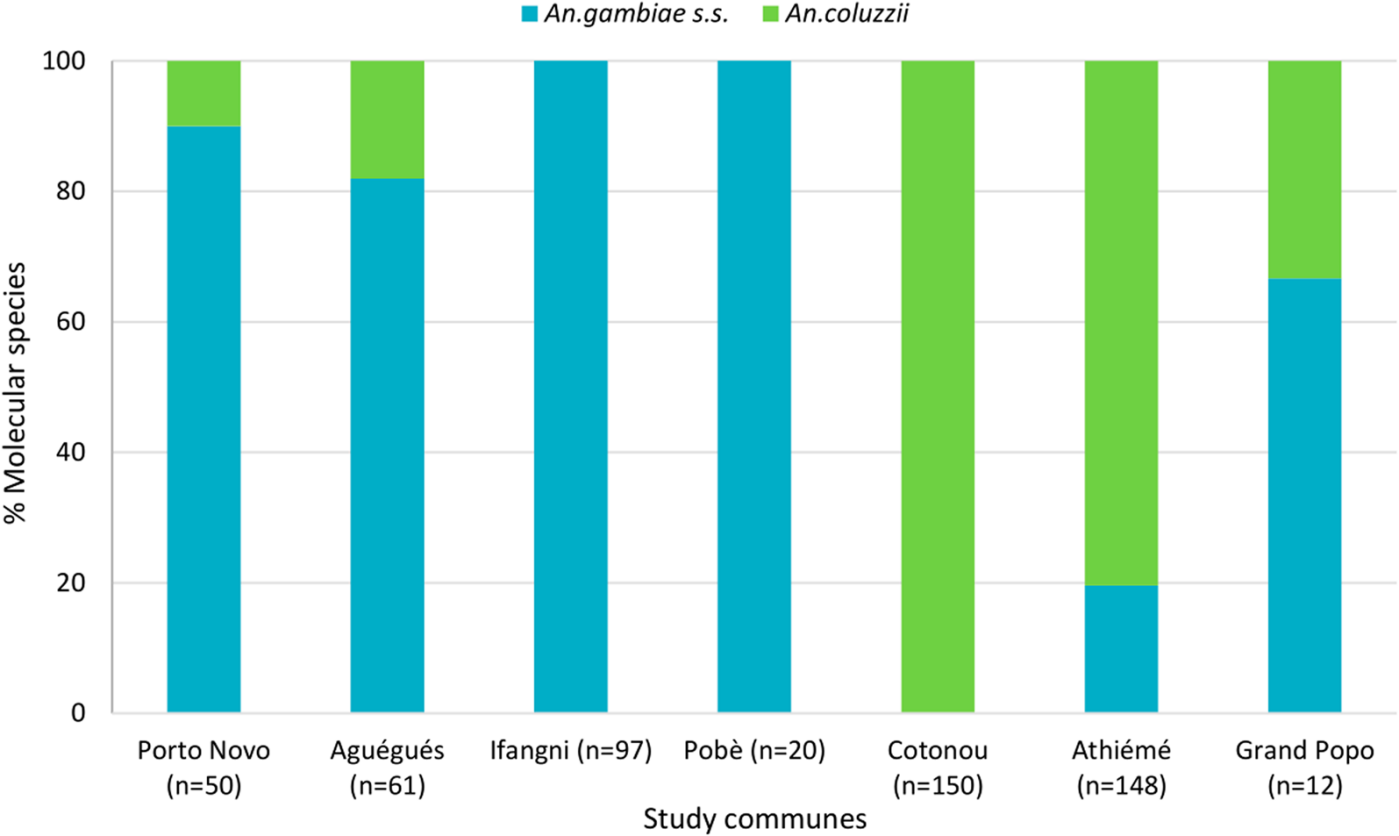
Proportions of molecular species in *An. gambiae s.l.* in study communes (HLC + PSC data)

Overall*,* the predominant species was *An. coluzzii* in Cotonou (100%), and Athiémé (80.4%), while it was *An. gambiae s.s.* in Porto-Novo, Aguégués, Ifangni, Pobè, and Grand-Popo with relative frequencies ranging between 66.7–100% (Fig. 3).

### Infection prevalence with *Wolbachia* and Microsporidia

**Fig. 4:**
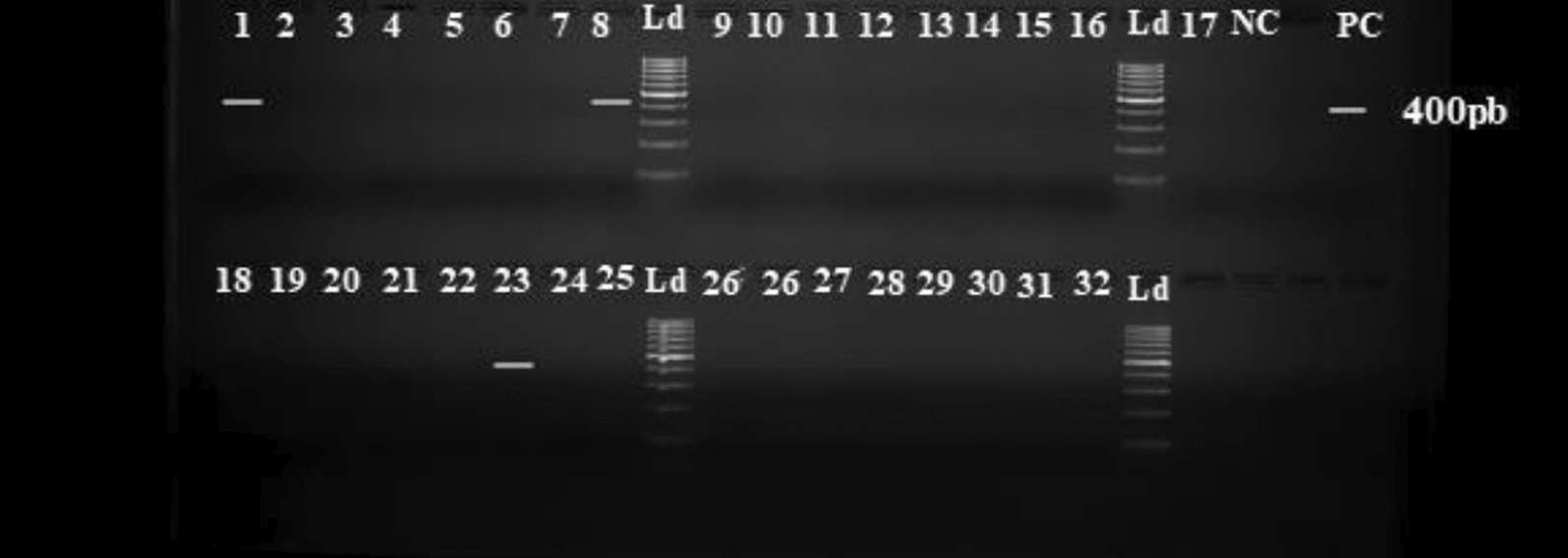
Results of 2% agarose gel electrophoresis of *Wolbachia* Anga 16S rDNA PCR (NC = Negative Control; PC = Positive Control; Ld = Ladder)

**Fig. 5:**
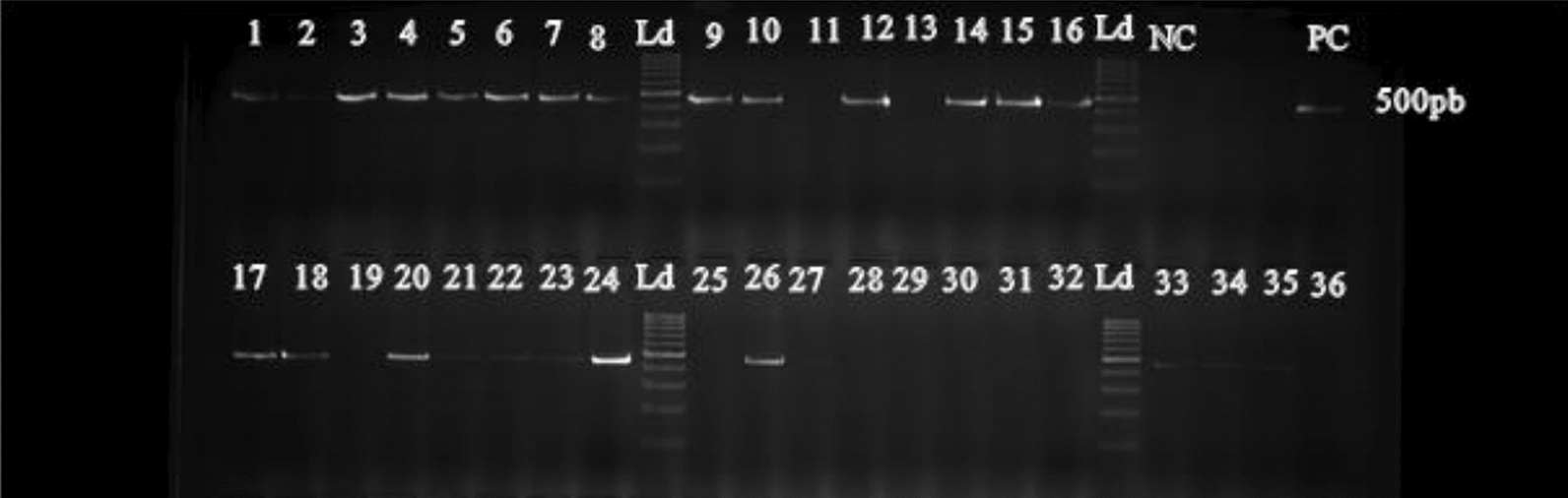
Results of 2% agarose gel electrophoresis of Microsporidia MB PCR (Ld = Ladder; NC = Negative Control; PC = Positive Control)

Overall, both *Wolbachia* (Fig. 4) and Microsporidia (Fig. 5) were identified in *An. gambiae s.l.* The infection prevalence with *Wolbachia* was 5.1% (95% CI 0.90–18.6) in *An. gambiae s.s. versus* 1.3% (95% CI 0.07–7.8) in *An. coluzzii* (p = 0.53), with a mean of 2.5% (95% CI 0.5–7.3) in the overall species complex (Table 1). Commune level data revealed the presence of *Wolbachia* in Ifangni, Pobè, and Cotonou (Additional file 1: Table S2).

**Table 1:** Infection prevalence with *Microsporidia MB* and *Wolbachia Anga* in the study area

Mosquito Species	N of pools	N (IP, 95% CI) *Wolbachia Anga*	N (IP, 95% CI) *Microsporidia MB*
*An. gambiae* s.s.	39	2 (5.1%, 0.90–18.6)	16 (41.0%, 25.9–57.8)
*An. coluzzii*	79	1 (1.3%, 0.07–7.8)	45 (57.0%, 45.4–67.9)
Grand total	118	3 (2.5%, 0.5–7.3)	63 (53.4%, 43.9–62.6)

*IP* infection prevalence, *CI* confidence interval

Infection prevalence with Microsporidia of 41.0% (95% CI 25.9–57.8) in *An. gambiae* s.s. *versus* 57.0% (95% CI 45.4–67.9) in *An. coluzzii* (p = 0.15), with a mean of 53.4% (95% CI 43.9–62.6) in the overall species complex was observed (Table 1). Irrespective of the molecular species, infection to Microsporidia was observed in all study communes (Additional file 1: Table S2).

### Sporozoite rate (SR) in *An. gambiae s.l.* and its molecular species

Of the 538 specimens of *An. gambiae s.l.* collected, 4 were infected (two from Cotonou, one from Porto-Novo and one from Aguégués), which equated to a mean SR of 0.74% (95% CI 0.20–1.90) (Table 2). At the molecular species level, the SR was 0.80% (95% CI 0.09–2.87) in *An. gambiae s.s. vs* 0.69% (95% CI 0.08–2.47) in *An. coluzzii* (p = 1). Of the four individual mosquitoes (two *An. coluzzii* and two *An. gambiae s.s*.), which harboured *P. falciparum*, none was infected with *Wolbachia* and one contained Microsporidia (*An. coluzzii*)*.*

**Table 2:** SR in *An. gambiae* s.l. and its molecular species

Mosquito species	N tested	N positive	SR (%)	95% CI
*An. gambiae* s.s.	249	2	0.80	0.09–2.87
*An. coluzzii*	289	2	0.69	0.08–2.47
*An. gambiae s.l.*	538	4	0.74	0.20–1.90

*N* number of *Anopheles*, *SR* sporozoite rate, *CI* confidence interval

The SR of each molecular species observed per study commune is detailed in Additional file 1: Table S3.

## Discussion

Given that the efficacy of insecticide-based control tools are under threat because of the emergence of resistance, there is a growing interest in the use of alternative, effective biological vector control strategies. For that, the search for natural endosymbiont-*Anopheles* systems capable of reducing vector competence has become essential. The present study is the first that reports the presence of *Wolbachia* and Microsporidia in both *An. gambiae s.s.* and *An. coluzzii* in Benin.

A trial recently conducted in Kenya showed that Microsporidia, a vertically transmitted bacteria was capable of disrupting *Plasmodium* development in *An. arabiensis* [22]*.* Moreover, it has been demonstrated that some mosquitoes can have their longevity reduced by *Wolbachia*, which prevents the completion of the life cycle of some infectious pathogens, thereby interrupting transmission [28]. Findings of the present study shows the natural presence of Microsporidia and *Wolbachia* in the microbiota of *An. gambiae s.l*. in Benin. These results confirm those of Gomes et al*.* [21] and Dada et al*.* [29] that demonstrated the ability of *An. gambiae s.l.* to host *Wolbachia* and Microsporidia.

In the present study, the infection rates (5.1% in *An. gambiae s.s.* and 1.3% in *An. coluzzii*) to *Wolbachia* was overall lower compared to those observed in Burkina-Faso (46% in *An. coluzzii* and 33% in *An. arabiensis*). The same trend was observed at the complex level (*An. gambiae s.l*.), with infection rates ranging between 46 and 78%, depending on the study site in Mali [21]. The general low infection prevalence of *Wolbachia* in the study area could be due to low density levels that were difficult to detect by PCR or reflect the insensitivity of the end-point PCR technique used. In a previous study in Mali, nested PCR failed to identify 21.7% of infected *An. gambiae s.l.* samples infected with *Wolbachia* wAnga-Mali with poor concordance between technical replicates, suggesting that *Wolbachia* levels were close to the limit of detection of these assays [21]. qPCR methodologies, recently developed for *Wolbachia* Anga, may have improved detection levels [21]; however, were not feasible with the limited laboratory resources. Thus, the null infection rate to *Wolbachia* observed in some study communes should not necessarily be interpreted as an absence of this endosymbiont. Taken together these results suggest that natural infection of *An. gambiae s.l.* to *Wolbachia* is highly variable across sites in Africa. A similar result was observed in China where the prevalence of *Wolbachia* natural infection was highly variable in field-collected mosquitoes (*Aedes albopictus*, *Anopheles sinensis*, *Armigeres subalbatus*, *Cx pipiens,* and *Culex tritaeniorhynchus*) collected across 25 surveyed provinces [30]. Moreover, *Wolbachia* natural infection could also be highly variable in various *Anopheles* species as previously reported in Gabon, Central Africa [31]. Of note, there is a huge diversity of *Wolbachia* strains with different effects in nature [19].

The deployment of a *Wolbachia*-based control tool for controlling mosquito borne diseases through the production of sterile insects or pathogen blocking, requires the inducement of cytoplasmic incompatibility to drive the bacterium into natural arthropod populations [32]. While *Wolbachia* has been shown to impact *P. falciparum* development, previous works revealed that *Wolbachia* detected in the present study do not confer cytoplasmic incompatibility [20] and, therefore, would not be feasible to use for control purposes.

The findings show a strong presence of Microsporidia in both *An. gambiae s.s.* and *An. coluzzii,* with a mean infection rate of 53.4%. This corroborates previous findings from Akorli et al*.* [33] who demonstrated that Microsporidia was highly associated with *An. gambiae s.s.* and *An. coluzzii* in Ghana. Also, a higher respective, albeit non-significant infection rate to Microsporidia was observed in *An. coluzzii* than in *An. gambiae* both in the present trial (57% vs 41%) and the one (80.7% vs 76.0%) of Akorli et al*.* [34]. Thus, one aspect worth investigating in future trials would be whether *An. coluzzii* is more susceptible to infection with Microsporidia, compared to *An. gambiae s.s.*

Though the present trial is a cross-sectional one, it is worth mentioning that investigating the dynamics or variations in bacterial diversity in field-collected adult populations of *An. gambiae s.l.* is challenging, as bacterial diversity is strongly influenced by several factors such as seasonality, locality-dependent acquisition of environmental microbes [34], diet at larval stage [35], sugar/blood feeding, mating [36], and other factors likely not yet studied.

Overall, in both HLCs and PSCs, the most frequent mosquito species collected were *Culex* spp, and *Mansonia* spp, followed by *Anopheles* spp, and *Aedes* spp. The same trend was previously observed in Cove, Ouinhi and Zangnanando communes located 156 km away from Cotonou, the economic capital of Benin [37]. Molecular species identification revealed the presence of a mixture of *An. coluzzii* and *An. gambiae s.s.* which is consistent with findings from several other previous trials conducted in Southern Benin [21, 35, 36]. Overall, the SR was similar in *An. gambiae s.s.* and *An. coluzzii*, which corroborates previous findings from Akogbeto et al*.* [38] in Northern Benin. However, given the *P. falciparum* infection rate was assessed at the mosquito level, while the infection rate to each endosymbiont was evaluated at the pool level, it was not possible to assess the influence of the presence of each endosymbiont on the *Plasmodium* sporozoite infection, which is a limitation for the study. Failure to carry out phylogenetic analyses in order to identify relationships between Microsporidia and *Wolbachia* detected in *An. gambiae s.l.* from Benin and those observed in other regions in Africa also constitutes another drawback of this study.

## Conclusion

The present study is the first to report the natural presence of both *Wolbachia* and Microsporidia in natural populations of *An. gambiae* in Benin. Sustained efforts should be made to widen the spectrum of bacteria identified in mosquitoes, with the potential to develop endosymbiont-based control tools; such interventions could be the game-changer in the control of malaria and arboviral disease transmission.

### Supplementary Information

**Additional file 1: Table S1.** Mosquito species composition (PSC data).** Table S2. **Infection prevalence with *Wolbachia Anga and Microsporidia MB *per molecular species (*An. gambiae* s.s. and *An. coluzzii*) in each study commune (HLC + PSC data). **Table S3. **SR per molecular species, in each study commune (HLC + PSC data).

## Data Availability

The data is available on reasonable request from the corresponding author.
