# Supplementary material for: First report of natural infection of Anopheles gambiae s.s. and Anopheles coluzzii by Wolbachia and Microsporidia in Benin: a cross-sectional study
Source: Malar J. 2024 Mar 11;23:72. doi: 10.1186/s12936-024-04906-1 (PMC10926679; doi:10.1186/s12936-024-04906-1)
Supplement: Supplementary file 1 — Additional file 1: Table S1. Mosquito species composition (PSC data). Table S2. Infection prevalence with Wolbachia Anga and Microsporidia MB per molecular species (An. gambiae s.s. and An. coluzzii) in each study commune (HLC + PSC data). Table S3. SR per molecular species, in each study commune (HLC + PSC data). [file 12936_2024_4906_MOESM1_ESM.docx]

**Additional file 1**

**Table S1: Mosquito species composition (PSC data)**

| Indicators/Mosquito species | *Anopheles gambiae s.l.* | *Aedes aegypti* | *Other Aedes* | *Culex quinquefasciatus* | *Other Culex* | *Mansonia africana* | Total |
| --- | --- | --- | --- | --- | --- | --- | --- |
| N | 28 | 22 | 6 | 197 | 7 | 45 | 305 |
| Frequency (%) | 9.2 | 7.2 | 2.0 | 64.6 | 2.3 | 14.8 | - |

N: number of collected mosquitoes

**Table S2: Infection prevalence with *Wolbachia Anga and Microsporidia MB* per molecular species (*An. gambiae* s.s. and *An. coluzzii*) in each study commune (HLC + PSC data).**

| Communes | Molecular Species | N | *Wolbachia Anga* | *Microsporidia MB* |
| --- | --- | --- | --- | --- |
|  |  | Pools | N Pools^+^ (IP) | N Pools^+^ (IP) |
| Porto-Novo | *An. gambiae* s.s*.* | 2 | 0 (0%) | 2 (100%) |
|  | *An. coluzzii* | 9 | 0 (0%) | 7 (77.8%) |
|  |  |  |  |  |
| Aguégués | *An. gambiae* s.s*.* | 3 | 0 (0%) | 1 (33.3%) |
|  | *An. coluzzii* | 10 | 0 (0%) | 4 (40%) |
|  |  |  |  |  |
| Ifangni | *An. gambiae* s.s*.* | 20 | 1 (5%) | 7 (35%) |
|  | *An. coluzzii* | 0 | 0 (n/a) | 0 (n/a) |
|  |  |  |  |  |
| Pobè | *An. gambiae* s.s*.* | 4 | 1 (25%) | 2 (50%) |
|  | *An. coluzzii* | 0 | 0 (n/a) | 0 (n/a) |
|  |  |  |  |  |
| Cotonou | *An. gambiae* s.s*.* | 0 | 0 | 0 (n/a) |
|  | *An. coluzzii* | 32 | 1 (3.1%) | 24 (75%) |
|  |  |  |  |  |
| Athiémé | *An. gambiae* s.s*.* | 8 | 0 (0%) | 2 (25%) |
|  | *An. coluzzii* | 26 | 0 (0%) | 10 (38.5%) |
|  |  |  |  |  |
| Grand Popo | *An. gambiae* s.s*.* | 2 | 0 (0%) | 2 (100%) |
|  | *An. coluzzii* | 2 | 0 (0%) | 2 (100%) |

HLC: human landing catches, PSC: Pyrethrum spray catches, N Pools^+^: number of positive pools, IP: Infection prevalence, n/a: no data

**Table S3: SR per molecular species, in each study commune (HLC + PSC data)**

| Communes | Molecular Species | N Tested | N Positive | SR (%) |
| --- | --- | --- | --- | --- |
| Porto-Novo | *An. gambiae* s.s. | 45 | 1 | 2.2 |
|  | *An. coluzzii* | 5 | 0 | 0.0 |
|  |  |  |  |  |
| Aguégués | *An. gambiae* s.s. | 50 | 1 | 2.0 |
|  | *An. coluzzii* | 11 | 0 | 0.0 |
|  |  |  |  |  |
| Ifangni | *An. gambiae* s.s. | 97 | 0 | 0.0 |
|  | *An. coluzzii* | 0 | 0 | n/a |
|  |  |  |  |  |
| Pobè | *An. gambiae* s.s. | 20 | 0 | 0.0 |
|  | *An. coluzzii* | 0 | 0 | n/a |
|  |  |  |  |  |
| Cotonou | *An. gambiae* s.s. | 0 | 0 | n/a |
|  | *An. coluzzii* | 150 | 2 | 1.3 |
|  |  |  |  |  |
| Athiémé | *An. gambiae* s.s. | 29 | 0 | 0.0 |
|  | *An. coluzzii* | 119 | 0 | 0.0 |
|  |  |  |  |  |
| Grand-Popo | *An. gambiae* s.s. | 8 | 0 | 0.0 |
|  | *An. coluzzii* | 4 | 0 | 0.0 |

N: number of *Anopheles*, SR: sporozoite rate, n/a: no data
